# Impact of Melatonin on Sepsis-Associated Acute Kidney Injury in Rat Model of Lipopolysaccharide Endotoxemia

**DOI:** 10.3390/cimb48010119

**Published:** 2026-01-22

**Authors:** Milan Potić, Ivan Ignjatović, Dragoslav Bašić, Ljubomir Dinić, Aleksandar Skakić, Zoran Damnjanović, Nebojša Jovanović, Milica Mitić, Dušan Sokolović

**Affiliations:** 1 Faculty of Medicine, University of Niš, 18000 Niš, Serbia; uropota@gmail.com (M.P.); ignjatovic1964@gmail.com (I.I.); basicdr@gmail.com (D.B.); ljubomidinic@gmail.com (L.D.); saleskaka@hotmail.com (A.S.); damnjanovicz@yahoo.com (Z.D.); 2 Clinic for Urology, University Clinical Center Niš, 18000 Niš, Serbia; 3 Clinic for Vascular Surgery, University Clinical Center Niš, 18000 Niš, Serbia; 4 Center for Gynecology and Human Reproduction, Medical Military Academy, 11000 Belgrade, Serbia; nebojsa038@gmail.com; 5 Center for Pathology and Pathological Anatomy, University Clinical Center Niš, 18000 Niš, Serbia; stankovic.milica93@gmail.com; 6 Institute for Treatment and Rehabilitation, Niška Banja, 18000 Niš, Serbia

**Keywords:** lipopolysaccharide, kidney, melatonin, apoptosis, inflammation, caspase

## Abstract

Sepsis-associated acute kidney injury (S-AKI) is a frequent and life-threatening condition, characterized by rapid functional decline, which is followed by intense inflammation and tissue injury. Experimental lipopolysaccharide (LPS)-induced sepsis reproduces functional and morphological features of human S-AKI and enables investigation of melatonin which has numerous beneficial properties, such as antioxidant properties. In this study, the effects of melatonin (50 mg/kg) on kidney dysfunction, oxidative damage, inflammation, apoptosis, and histopathological alterations in a rat model of S-AKI induced by LPS application (10 mg/kg) were studied. Acute LPS exposure caused statistically significant (*p* ≤ 0.05) marked renal dysfunction, increased lipid and protein oxidation, suppression of antioxidant enzymes, enhanced NO/iNOS signaling, elevated pro-inflammatory cytokines (TNF-α, IL-1β, IL-6), activation of apoptotic pathways, and pronounced tubular and glomerular injury. Co-administration of melatonin statistically significantly (*p* ≤ 0.05) attenuated oxidative stress, reduced production of inflammatory cytokines, suppressed apoptosis, and ameliorated structural kidney damage, leading to partial restoration of renal function. These findings suggest that melatonin exerts renoprotective effects in S-AKI through combined antioxidant, anti-inflammatory, and anti-apoptotic actions, likely involving modulation of different signaling pathways.

## 1. Introduction

Sepsis-associated acute kidney injury (S-AKI) is one of the most frequent and lethal organ dysfunctions in critically ill patients. Some studies suggest that up to two-thirds of patients with septic shock develop AKI, increasing the risk for mortality [1,2,3]. Experimental models analyzing the mechanisms associated with S-AKI are of great importance, which is especially true for the mechanisms that are hard or impossible to be studied in patients. One such model is Gram-negative bacteria lipopolysaccharide (LPS)-induced sepsis, with consequential decline in renal function. In this model, a rapid change in serum kidney damage parameters can be seen, which is followed by a reduced urine output [4,5] and micromorphological alterations mirroring key structural hallmarks reported on biopsies obtained from patients with S-AKI [1,6].

In this model, the renal injury arises from a complex interaction between oxidative stress, inflammation, and cell-death-associated pathways. In the presence of LPS, there is a strong reaction in tubular epithelial and endothelial cells, which is activated through Toll-like receptor 4 (TLR4), leading to an early inflammatory response and increased production of cytokines (TNF-α, IL-1β, and IL-6) and chemokines involved in inflammatory cell recruitment [3,6]. Also, renal tissue shows increased myeloperoxidase activity and marked leukocyte infiltration [7]. In parallel with inflammation, oxidative stress causes additional tissue damage by affecting lipids, proteins, and DNA, causing cell damage and dysfunction that reflects on organ dysfunction itself. This occurs through disruption of mitochondrial function, excess production of reactive oxygen and nitrogen species (ROS and RNS), and disturbances in antioxidant enzymes such as catalase (CAT) and superoxide dismutase (SOD) [8]. Inflammation and oxidative damage drive cells to different death pathways, one of which is programmed cell death, apoptosis. In the LPS-induced S-AKI model, there is an increased Bax/Bcl-2 ratio, cytochrome c release, and activation of executioner caspases, which are known mediators and executors of apoptosis [9].

Melatonin (N-acetyl-5-methoxytryptamine, MLT) is an indoleamine secreted primarily by the cells located in the pineal gland, as well as some other cells, that exerts potent antioxidant, anti-inflammatory, and anti-apoptotic actions. MLT actions are associated with distinct signaling pathways and are likely mediated by receptors and by direct MLT action. Previous publications indicate that MLT may possess protective effects in LPS-induced S-AKI [10,11,12]; however, the mechanisms underlying its action remain unclear and largely unknown and uninvestigated. Apart from the kidney, MLT alleviates septic shock accompanied by body responses in rats exposed to either LPS or polymicrobial pathogens [11,13]. Some in vitro actions of MLT involve mitochondrial function stabilizing potential, interference with nuclear factor erythroid 2-related factor 2 (Nrf2) signaling, and free radical scavenging [11,13] which could contribute to its activity in the model of S-AKI.

The aim of the present study was to investigate the protective effects of MLT application in a rat model of LPS-induced sepsis-associated acute kidney injury, with a particular focus on renal function, oxidative and nitrosative stress, inflammatory responses, apoptosis-related pathways, and histopathological alterations, in order to elucidate the potential mechanisms underlying MLT’s action.

## 2. Materials and Methods

### 2.1. Chemicals

Lipopolysaccharide (LPS from Escherichia coli serotype O111:B4; Sigma–Aldrich, St. Louis, MO, USA) was freshly prepared in sterile physiological saline (0.9% NaCl) and administered intraperitoneally at a dose of 10 mg/kg. Melatonin (Sigma–Aldrich) was dissolved in 8% ethanol in physiological saline (0.9% NaCl) to achieve the required concentration and was administered by oral gavage at a dose of 50 mg/kg.

### 2.2. Animals and Experimental Design

Adult male Wistar rats (250–300 g) were housed under conventional laboratory conditions (22 ± 1 °C, relative humidity 55–65%, 12 h light/dark cycle). Standard laboratory chow and tap water were available ad libitum. All experimental procedures followed national regulations governing the use of laboratory animals and were carried out after the local Ethics Committee approved the current study. All experimental procedures were performed in accordance with the ethical regulations of the Helsinki and European Community guidelines for the ethical handling of laboratory animals (EU Directive of 2010; 2010/63/EU), as well as those given by the laws of the Republic of Serbia (decision number: 323–07–01762/2021–01 from 13 May 2021).

The study design followed that of previous publications, using the doses and routes of application for both LPS [10,14] and MLT [15]. Twenty-eight Wistar albino rats were randomly allocated into four groups, each consisting of six animals:

Group I—Control group—rats received vehicle (8% ethanol in 0.9% NaCl, 10 mL/kg) by oral gavage.

Group II—Melatonin (MLT) group—rats were given a single oral dose of melatonin (50 mg/kg) in vehicle.

Group III—LPS group—rats received a single i.p. injection of LPS (10 mg/kg) and oral vehicle.

Group IV—LPS + MLT group—rats were pretreated with a single oral dose of melatonin (50 mg/kg), followed by a single i.p. injection of LPS (10 mg/kg).

Twelve hours after treatment, the animals were anesthetized with an overdose of ketamine and euthanized. Blood samples were obtained from the abdominal aorta, allowed to clot at room temperature, and centrifuged at 1500 rpm for 10 min to separate serum. Both kidneys were rapidly excised, rinsed in ice-cold physiological saline, and processed further. One kidney was fixed in buffered formalin for histopathological examination, while the contralateral kidney was snap-frozen in liquid nitrogen and stored at −80 °C for subsequent biochemical analyses. Kidney tissue homogenates were prepared in phosphate buffer and centrifuged at 10,000 rpm for 15 min at 4 °C to obtain clear supernatants. The resulting supernatants were used for protein quantification according to the Lowry method [16] and subsequently employed for biochemical measurements.

### 2.3. Serum Biochemical Analysis

Serum levels of urea and creatinine and concentrations of sodium and potassium were measured using an automated biochemical analyzer (Olympus AU680, Olympus, Tokyo, Japan). Measurements were performed in duplicate, and mean values were used for statistical analysis.

### 2.4. Tissue Biochemical Analyses

#### 2.4.1. Oxidative Stress Parameters and Antioxidative Potential Determination

The extent of lipid peroxidation was estimated through measurement of thiobarbituric reactive substances (TBARSs) in a reaction between lipid products in tissue homogenate and thiobarbituric acid under acidic conditions [17]. The results were calculated using a molar coefficient and expressed as μmol/mg protein. Advanced oxidation protein products (AOPPs) were assessed by determining carbonyl group content in a derivatization reaction with dinitrophenylhydrazine under acetic conditions [18]. AOPP concentrations were calculated using the molar extinction coefficient of DNPH and results are expressed as μmol/mg of tissue proteins.

Catalase (CAT) activity in kidney tissue was assessed by quantifying the reaction product formed between the enzyme present in tissue homogenates, hydrogen peroxide (H_2_O_2_) as the substrate, and ammonium molybdate [14]. Enzyme activity was expressed as units per milligram of kidney tissue protein (U/mg protein). Superoxide dismutase (SOD) activity was determined using a colorimetric assay kit (Abcam, ab65354, Cambridge, UK), based on the reaction of water-soluble tetrazolium salt (WST) with superoxide anions generated in the tissue homogenate, with absorbance measured at 450 nm. SOD activity was expressed as the percentage inhibition of the reaction, in accordance with the manufacturer’s instructions.

#### 2.4.2. Nitric Oxide and iNOS Determination

Tissue NO (nitrite and nitrate) content was measured using a Griess reaction. After deproteinization, equal volumes of supernatants were incubated with sulfanilamide and N-(1-naphthyl)ethylenediamine dihydrochloride, and the absorbance was measured at 540 nm [19]. Concentrations were expressed as μmol/mg protein.

The iNOS amount in kidney tissue was determined using an ELISA (CUSBIO, CSB-E08325r, Houston, TX, USA) sandwich enzyme-linked immunosorbent assay kit. The obtained results are given as ng/mg of tissue proteins.

#### 2.4.3. Quantification of Inflammatory Mediators

The concentrations of tumor necrosis factor-α (TNF-α; Rat TNF alpha ELISA Kit Abcam, Boston, MA, USA; ab236712), interleukin-1β (IL-1β; Rat IL-1β/IL-1F2 Quantikine ELISA, R&D Systems, Minneapolis, MN, USA; RLB00), and interleukin-6 (IL-6; Quantikine ELISA Rat IL-6, R&DSystems, Minneapolis, MN, USA; R6000B) in renal tissue homogenates were analyzed using specific ELISA kits. Samples and standards were run in parallel, and cytokine levels were calculated from calibration curves, and the final results are presented as pg/mg of total protein.

#### 2.4.4. Apoptosis-Related Parameter Determination

Tissue alkaline and acid DNase activity were measured using DNA, a substrate, and buffers of different acidity mimicking the optimal pH for enzyme action [20]. For alkaline DNase I the optimum pH buffer (Tris-HCl) for reaction was 7.4, while for the acid DNase II activity the optimum pH of acetate buffer was 5.0. The activity of the enzyme was expressed as mU/mg of protein.

The amounts of caspase-3 (CUSABIO, CSB-E08857r, Houston, TX, USA) and caspase-9 (CUSABIO, CSB-E08863r, Houston, TX, USA) were determined using appropriate ELISA kits. The obtained results are given as ng/mg of tissue proteins.

### 2.5. Histopathological Analysis

Formalin-fixed (10%, *w*/*v*) kidney tissue underwent dehydration using ethanol solutions at varying concentrations (50–100%, *v*/*v*) and was finally embedded in paraffin. Tissue sections, cut into 4–5 μm thick pieces, were further stained with the standard histochemical technique of hematoxylin and eosin (HE). Microscopic examination, by a researcher blind to the treatment (M.M.), was performed using an Olympus BH2 light microscope (Olympus America Inc., Valley, PA, USA). Tissue examination included changes in the glomerular spaces and tubular structures. The evaluation of kidney morphological changes involved a grading system: 0 (no change), 1 (mild changes; <30%), 2 (moderate changes; 30–50%), and 3 (severe changes; >50%) [21].

### 2.6. Statistical Analysis

The obtained results are expressed as mean ± standard deviation and, in the case of morphological changes, only as mean values. The statistical differences between the groups were evaluated using ANOVA followed by Tukey’s post hoc test. Statistical significance was set at *p* ≤ 0.05. All statistical analyses were performed using GraphPad Prism (version 8.0).

## 3. Results

### 3.1. Renal Function

Twelve hours after the application of LPS a statistically significant (*p* < 0.001) alteration in all measured kidney functional parameters was noted (Table 1). In the group that received MLT together with LPS the values of urea and potassium were significantly increased (*p* < 0.01), compared to the control, while the values of creatinine and sodium were in the range of healthy rats and significantly lower than in the LPS-treated rats (*p* < 0.01) (Table 1).

### 3.2. Tissue Oxidative Stress

**Figure 1:**
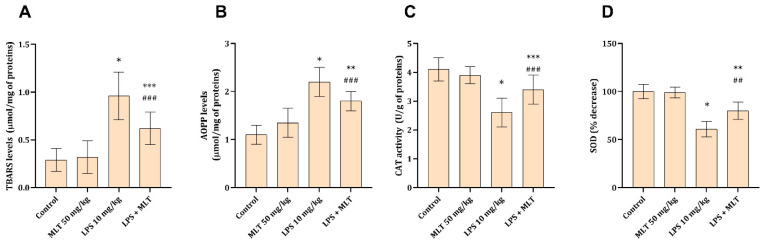
Kidney tissue oxidative stress parameters ((**A**) TBARS and (**B**) AOPP) and antioxidative potential ((**C**) CAT and (**D**) SOD) obtained from rats belonging to different experimental groups. Data are shown as mean ± SD (n = 6). One-way ANOVA, followed by Tukey’s post hoc test, * *p* < 0.001, ** *p* < 0.01, *** *p* < 0.05 vs. control; ## *p* < 0.01, ### *p* < 0.05 vs. LPS-treated animals.

The kidney levels of TBARSs and AOPPs were found to be significantly increased (*p* < 0.001) in rats exposed to LPS (Figure 1A,B), only 12 h after the exposure to endotoxin. Application of MLT together with LPS had a moderate impact on LPS-induced oxidative protein and lipid damage, causing slight statistically significant (*p* < 0.05) decreases in AOPPs and TBARSs in the examined tissue (Figure 1A,B).

The antioxidative tissue capacities, estimated through the CAT and SOD activities, were found to be statistically significantly decreased (*p* < 0.001) in kidney tissue of animals exposed to LPS (Figure 1C,D). Co-application of MLT with LPS led to an improvement in both studied antioxidant enzyme activities, with slightly higher impact on SOD activity (*p* < 0.01) (Figure 1C,D).

### 3.3. Kidney Tissue Inflammation

**Figure 2:**
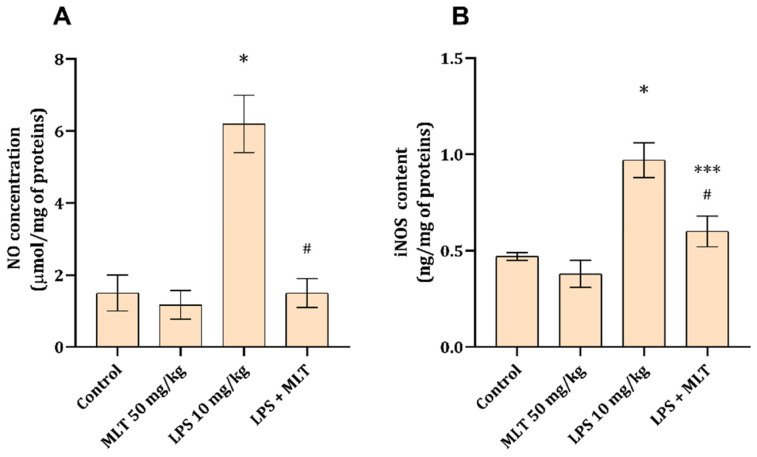
NO (**A**) and iNOS (**B**) contents in rat kidney tissue obtained from different experimental groups. Data are shown as mean ± SD (n = 6). One-way ANOVA, followed by Tukey’s post hoc test, * *p* < 0.001, *** *p* < 0.05 vs. control; # *p* < 0.001 vs. LPS-treated animals.

Kidney tissue NO and iNOS contents were found to be statistically significantly increased (*p* < 0.001), compared to the control, in animals that received LPS (Figure 2A,B). Rats treated with MLT and LPS at the same time had significantly (*p* < 0.001) lower amounts of NO and iNOS (Figure 2A,B), and the impact on NO concentrations was more pronounced than that on iNOS content.

Application of LPS to rats led to a statistically significant (*p* < 0.001) increase in kidney tissue pro-inflammatory parameters, i.e., TNF-α, IL-1β, and IL-6 (Table 2), with an almost 10-fold increase in each of them. Application of MLT and LPS significantly prevented an increase in the studied parameters, with 5-fold and 2-fold increases in TNF-α (*p* < 0.01) and IL-1β (*p* < 0.05), respectively (Table 2). Interestingly, there was no observable statistically significant (*p* > 0.05) increase in IL-6 levels in rats treated with LPS and MLT, in comparison to the values in the control group (Table 2).

### 3.4. Tissue Apoptosis

Acute exposure of rats to LPS lead to statistically significant (*p* < 0.001) increases in kidney tissue apoptosis-associated parameters, caspase-9, caspase-3, and acidic and alkaline DNAase (Table 2). Co-administration of MLT with LPS significantly (*p* < 0.001) prevented increases in the studied parameters associated with apoptosis (Table 2).

### 3.5. Kidney Tissue Histopathology

**Figure 3:**
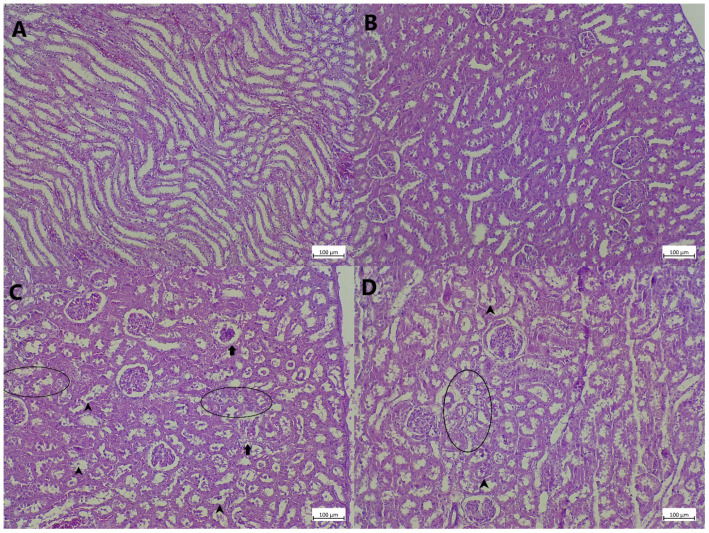
Histopathological analysis of rat kidney tissue stained with HE (magnification is ×100). In rats belonging to control and MLT group ((**A**) and (**B**), respectively) regular kidney tissue structures were observed. (**C**) In animals exposed to LPS, glomerular changes (arrow), tubular degeneration and intraluminal content (arrowhead), together with inflammatory infiltration (circled), were observed; (**D**) animals treated with MLT and LPS where minimal changes in the kidneys in the form of degeneration and occasional tubular deposit (arrowhead) and mild inflammatory response (circled) were seen.

Kidney tissue of animals belonging to control and MLT-treated groups were without any detectable microscopic alterations, with normal sized glomeruli and unaltered tubular system, with no intraluminal material present, and with no inflammatory infiltrate (Figure 3A,B, Table 3). In the kidney tissue of animals treated with LPS only moderate/severe alterations in glomerular size were observed, with occasional collapse of the glomerular tuffs (Figure 3C). Intense and widespread tubular degeneration and cloudy swelling, with prominent intraluminal content, could be seen (Figure 3C, Table 3). Finally, prominent peritubular and periglomerular inflammatory cell infiltrate was observed in kidney tissue of animals belonging to this group (Figure 3C, Table 3). In animals treated with MLT together with LPS almost identical changes to the ones observed in LPS-treated rats were seen, however, the extent of the changes and their presence was milder (Figure 3D, Table 3).

## 4. Discussion

Animals were sacrificed 12 h after the induction of endotoxemia, which corresponds to the acute phase of LPS-induced sepsis. Melatonin (MLT) attenuated S-AKI by preserving renal function and morphology through simultaneous suppression of oxidative stress and nitric oxide signalization, inhibition of inflammatory signaling and cytokine production, and reduction of tubular cell apoptosis via modulation of multiple interconnected molecular pathways. During this period, a marked deterioration in renal function was observed, reflected by increased serum urea, creatinine, and potassium levels, accompanied by reduced sodium concentrations (Table 1). This result implies that S-AIK occurred and that the kidney tissue suffered [10,22]. The changes at the level of glomeruli in LPS-treated rats (Figure 3C and Table 3) can be linked to the structural alterations reported in human S-AKI biopsies [1,6]. Alterations in serum biochemical parameters, including azotemia and electrolyte imbalance, correlated with morphological tissue damage, such as tubular necrosis and intratubular casts [23], as observed in the animals receiving LPS only (Figure 3C). In some previous experimental settings MLT has been proven to prevent significant alterations in serum kidney functional markers [10], however, only speculative suggestions associated with its mechanism of action, as a potential way to prevent cell membrane damage, were given [24].

Oxidative damage, evidenced by elevated TBARS and AOPP levels, in rats exposed to LPS is often considered one of the initial steps in S-AKI that further progresses into irrecoverable cell damage and death [4]. An imbalance between ROS and cellular antioxidant capacities leads to the accumulation of oxidative products from lipids and proteins. The end products of lipid peroxidation, presented as TBARSs in the present study, are known to alter cell membrane properties, causing its deformation and impairment in function (altered ion transport and enzymatic activity) [25]. Similarly, AOPPs, as markers of protein oxidative modification, correlate with cellular dysfunction and inhibition of enzymatic systems [26]. At the microscopic level, acute tubular injury, seen as tubular dilatation, epithelial cell swelling, loss of brush border, and casts in the tubular lumen, are just some hallmarks of extensive ROS formation and their devastating consequences [4,5]. The role of MLT in preventing ROS generation and their consequential damage to the described molecules has been debated many times and is related to the molecules’ ability to directly scavenge free radicals [14]. The role of antioxidant enzymes, CAT and SOD, is in prevention of the devastating damage by ROS to cell building molecules and in blocking inflammatory cytokine signals and endotoxin transduction [27]. Their impaired activity is seen in numerous kidney disorders, including sepsis [4]. The ability of MLT to increase, or prevent a decrease, in CAT and SOD activity is either through ROS scavenging or through the induction of their synthesis via Nrf2 activation [12].

One of the key pathophysiological mechanisms that mediates LPS-associated hemodynamic changes, as seen in S-AKI, is the NO/iNOS system [28] which generates NO from arginine in the presence of iNOS [29]. Excessive NO production following LPS exposure disrupts signaling pathways, impairs renal hemodynamics, and promotes inflammatory responses [30]. Also, tubular degeneration and apoptosis, as observed in the animals treated with LPS only (Figure 3C), have been shown to be the consequence of RNS formation and their direct devastating impact [31]. Melatonin supplementation has been shown to reduce iNOS expression in various models of kidney damage and is potentially associated with reduced p38-mitogen-activated protein kinase (MAPK) [32]. Thus, a diminished NO production and decreased iNOS amounts (Figure 2A,B) and reduced tubular damage (Figure 3D) seen in rats exposed to MLT together with LPS can be potentially attributed to MLT action on p38-MAPK as previously suggested [32]. Also, since the main sources of iNOS are inflammatory cells one can correlate a decrease in iNOS with the ability of MLT to impede the function and infiltration of macrophages and neutrophils [33], which was noted in the reduction in inflammatory cell infiltrate (Figure 3D and Table 3).

The immune system response not only mediates early host defense against infective agents but also significantly contributes to the development of septic shock and S-AKI [4]. The inflammatory response involves inflammatory cell recruitment (activation and infiltration) and production of a milieu of cytokines targeting other immune system cells and organ-specific cells. LPS administration results in increased infiltration of inflammatory cells around glomerular and tubular structures (Figure 3C and Table 3), as well as increases in the measured concentration of pro-inflammatory cytokines in kidney tissue (Table 2). One of the key steps in the inflammatory response to LPS is the activation of the cytoplasmic NOD-like receptor protein 3 (NLRP3) inflammasome, which activates caspase-1 that further assists in the formation of IL-1β [6] that has been found in kidney tissue of animals exposed to LPS (Table 2). Also, TNF-α is believed to be directly involved in progression of S-AKI, since its levels correlate with sepsis intensity. The activation of TNF-α on the endothelial cells in glomerular blood vessels is just one of the mechanisms through which this cytokine impairs kidney functioning [34]. The role of IL-6 in sepsis and S-AKI is mainly associated with the intensity of immune cell infiltration and their interaction and activation in defense towards pathogens [35]. Regarding the impact of MLT on decreasing IL-1β levels, it might be possible through the previously shown mechanism involving prevention of NLRP3 inflammasome formation and caspase-1 generation [36]. The possible mechanisms through which MLT could impact the production of the studied cytokines is through the blockage of nuclear translocation and DNA binding of the NF-κB p50 subunit, thus preventing cytokine production downstream [37] as well as the interference with the phosphoinositide 3-kinase/protein kinase B (PI3K/AKT) signaling pathway [38]. These are just some of the potential mechanisms deserving further studies and explanations and giving a plausible mode of action for MLT. Importantly, therapeutic strategies targeting single inflammatory mediators, such as anti-TNF-α antibodies, have failed in clinical sepsis [34], highlighting the potential advantage of agents like MLT that simultaneously modulate multiple signaling pathways.

It is well known that extensive oxidative damage and inflammatory cell response and signalization ultimately promote cell death, predominantly apoptosis. In addition to classical apoptosis, more recently described forms of regulated cell death (such as pyroptosis mediated by inflammasome–caspase-1–gasdermin D signaling and necroptosis) also contribute to both experimental and clinical tubular injury in S-AKI [39]. The results of the present study support that apoptosis as a dominant mechanism of tubular cell loss (Table 3 and Figure 3), driven by severe oxidative tissue damage (Figure 1A,B) and robust inflammatory response (Table 2), is indeed one of the dominant mechanisms associated with tubular cell death in S-AKI. Mitochondrial dysfunction and increased membrane permeabilization lead to cytochrome c release, that furthers forms apoptosome complexes and activates caspase-9 [9]. Such activated caspase cleaves and activates a central effector of apoptosis, caspase-3, which down the line activates lysosomal and nuclear DNases, i.e., DNase I and DNase II, respectively [9]. The described molecular changes reflect the microscopic tissue with a dominance of altered (swollen) tubular cells and massive luminal debris in rats treated with LPS only (Figure 3C and Table 3). The protective activity of MLT in partial suppression of apoptosis in the present experiment can be explained through several mechanisms. The first one involves the ability of MLT to diminish TLRs that mediate LPS-associated apoptosis and caspase-3 activation through reduced TLR protein expression and mRNA levels [40]. The second mechanism might involve peroxidase proliferative receptor α (PPARα) activation and prevention of caspase-9 activation and expression, as have previously been shown in the model of tubular cell apoptosis [41]. Finally, overall action of MLT on the inhibition of ROS and NO formation, two factors associated with apoptosis in sepsis [42], generally contributes to better understanding of its potential via affecting multiple signaling pathways.

There are some limitations of this study that should be acknowledged. First, the experimental model relied on LPS-induced endotoxemia, which reproduces key features of S-AKI but does not fully capture the complexity and heterogeneity of clinical sepsis in humans. Second, all analyses were performed at a single early time point (12 h), limiting insight into the temporal progression of sepsis and long-term renal outcomes. Third, although multiple biochemical, histological, and molecular parameters were assessed, the study lacks confirmations based on pathway-specific inhibitors or genetic approaches. Fourth, only one dose and administration of MLT was examined, precluding dose–response evaluation and optimization of therapeutic timing. However, the study points to future research potential and hints at the possible study designs. These findings support the potential clinical use of MLT as an adjunctive, multi-target therapeutic strategy in S-AKI, warranting future dose-optimized and time-dependent clinical trials to evaluate its renoprotective efficacy and safety in septic patients.

## 5. Conclusions

The present study demonstrates that lipopolysaccharide-induced sepsis causes severe functional impairment and structural damage of the kidney, driven by excessive oxidative and nitrosative stress, pronounced inflammatory activation, and induction of apoptosis. Although the application of melatonin to rats together with LPS diminished changes in serum parameters and tissue oxidative damage, antioxidant capacity, nitric oxide signaling, inflammation, and apoptosis parameters, still mild morphological changes were visible. This indicates that the ability of melatonin to prevent sepsis-induced kidney damage is not absolute and is still limited. These findings position melatonin, with its numerous beneficial properties, as a promising candidate for further translational research aimed at preventing or attenuating sepsis-associated kidney injury.

## Figures and Tables

**Table 1:** Biochemical serum parameters in animals belonging to different experimental groups.

Parameter	Control	MLT	LPS	LPS + MLT
**Urea** **levels (mmol/L)**	5.1 ± 0.9	5.8 ± 0.7	14.2 ± 2.3 *	9.8 ± 1.5 **^,#^
**Creatinine levels (mg/dL)**	0.42 ± 0.05	0.45 ± 0.07	1.16 ± 0.2 *	0.54 ± 0.1 ^#^
**Sodium (Na^+^) levels (mmol/L)**	142 ± 5	145 ± 4	132 ± 3 **	139 ± 2 ^##^
**Potassium (K^+^) levels (mmol/L)**	4.4 ± 0.2	4.5 ± 0.3	6.1 ± 0.4 *	5.3 ± 0.3 **^,##^

Data are shown as mean ± SD (n = 6). One-way ANOVA, followed by Tukey’s post hoc test, * *p* < 0.001, ** *p* < 0.01 vs. control; ^#^ *p* < 0.001, ^##^ *p* < 0.01 vs. LPS-treated animals.

**Table 2:** Inflammation and apoptosis-associated parameters in kidney tissue obtained from rats belonging to different experimental groups.

Parameter	Control	MLT	LPS	LPS + MLT
TNF-α (pg/mg of proteins)	20.5 ± 4.7	25.1 ± 5.9	273 ± 36.4 *	117 ± 11.8 **^,#^
IL-1β (pg/mg of proteins)	9.1 ± 3.3	12.8 ± 4.2	89.9 ± 10.5 *	25.6 ± 9.8 ***^,#^
IL-6 (pg/mg of proteins)	1.1 ± 0.13	0.49 ± 0.26	10.83 ± 1.6 *	2.94 ± 1.2 ^#^
DNase I (mU/mg of proteins)	0.23 ± 0.08	0.16 ± 0.09	0.65 ± 0.1 *	0.32 ± 0.12 ^#^
DNase II (mU/mg of proteins)	0.56 ± 0.1	0.48 ± 0.12	1.25 ± 0.21 *	0.71 ± 0.20 ^#^
Caspase-3 (ng/mg of proteins)	0.17 ± 0.02	0.07 ± 0.03	0.41 ± 0.04 *	0.15 ± 0.07 ^#^
Caspase-9 (ng/mg of proteins)	0.73 ± 0.19	0.68 ± 0.12	1.93 ± 0.23 *	0.98 ± 0.31 ^#^

Data are shown as mean ± SD (n = 6). One-way ANOVA, followed by Tukey’s post hoc test, * *p* < 0.001, ** *p* < 0.01, *** *p* < 0.05 vs. control; ^#^ *p* < 0.001 vs. LPS-treated animals.

**Table 3:** Morphological changes in kidneys of animals belonging to different experimental groups.

Parameter	Control	MLT	LPS	LPS + MLT
**Glomerular** **degeneration**	0	0	2.1	0.7
**Tubular swelling**	0	0	2.9	1.1
**Tubular casts**	0	0	2.2	0.9
**Inflammation**	0	0	2.5	1.3

Grading: 0 (no change), 1 (mild changes; <30%), 2 (moderate; 30–50%), and 3 (severe changes; >50%).

## Data Availability

The original contributions presented in this study are included in the article. Further inquiries can be directed to the corresponding author.

## Abbreviations

The following abbreviations are used in this manuscript:

AOPP	advanced oxidation protein product
AKI	acute kidney injury
CAT	catalase
DNase	deoxyribonuclease
ELISA	enzyme-linked immunosorbent assay
HE	hematoxylin and eosin
IL-1β	interleukin-1 beta
IL-6	interleukin-6
iNOS	inducible nitric oxide synthase
LPS	lipopolysaccharide
MAPK	mitogen-activated protein kinase
MLT	melatonin
NLRP3	NOD-like receptor protein 3
NO	nitric oxide
Nrf2	nuclear factor erythroid 2-related factor 2
PI3K	phosphoinositide 3-kinase
RNS	reactive nitrogen species
ROS	reactive oxygen species
S-AKI	sepsis-associated acute kidney injury
SOD	superoxide dismutase
TBARS	thiobarbituric acid reactive substance
TLR4	Toll-like receptor 4
TNF-α	tumor necrosis factor alpha
